# Establishing the optimal body mass index - body esteem relationship in young adolescents

**DOI:** 10.1186/1471-2458-13-662

**Published:** 2013-07-17

**Authors:** Michael J Duncan, Yahya al-Nakeeb, Alan, M Nevill

**Affiliations:** 1 Department of Biomolecular and Sports Sciences, Coventry University, Priory Street, Coventry CV1 5FB, UK; 2 Qatar University, Doha, Qatar; 3 University of Wolverhampton, Wolverhampton, UK

**Keywords:** Obesity, Body image, Ethnicity, Body mass index, Inverted body mass index, Leanness

## Abstract

**Background:**

This study sought to compare the utility of either inverted body mass index or body mass index to optimise the relationship with body esteem in young adolescents Design: The study was cross sectional in design and assessed body esteem and weight status in756 young adolescents (394 boys, 362 girls, mean age *±* S.D. 11.4 *±* 1.6 years).

**Methods:**

Body esteem was determined using the body esteem scale for children. Height and body mass were measured directly. Body mass index was determined as kg/m^2^ and iBMI as cm^2^/kg.

**Results:**

Results indicated that the association between iBMI and body esteem was curvilinear in nature and iBMI was the better predictor of body esteem (P = .001) predicting 21.3% of the variance in body esteem scores compared to 20.5% using BMI (P = .001). When split by gender, the curvilinear relationship was still evident but significantly different between boys and girls although iBMI remained a better predictor of body esteem compared to BMI in both boys and girls. The peak differed between gender groups with the association between iBMI and body esteem peaking at 642 cm^2^/kg for boys and 800.64 cm^2^/kg for girls.

**Conclusion:**

This study suggests that iBMI is a better predictor of body esteem in young adolescents, and that the association between body esteem and iBMI is curvilinear in nature. However, the peak of body esteem scores occured at a lower degree of leanness for boys compared to girls and indicated that the point at which body esteem scores are highest for girls is at a point of extreme leanness whereas the peak for boys was within the values considered as ‘normal’ on the leanness to obesity continuum. iBMI may therefore be a useful measure of leanness for future studies examining the association between overweight/obesity and body esteem in young adolescents.

## Background

Overweight and obesity have been identified as a significant influence on a range of variables in children and adolescents including physical activity [1], metabolic abnormality [2] and psychological well-being [3]. In the case of the latter, body image concerns in childhood are becoming an increasingly prevalent indicator of poorer psychological well-being in western society. Such concerns have been linked to a number of social, psychological and physiological problems including increased incidence of eating disorders, restricted eating, poor psychological well being, obesity and excessive exercise [4,5]. Overweight and obesity have been identified as particularly important in the development of these concerns [6,7] and a number of studies have examined the relations between various measures of body image and overweight/obesity in children, adolescents and adults [7-11].

For example, Body Mass Index (BMI) has typically been employed as a surrogate measure of obesity in body image research with some studies identifying higher BMI scores being associated with more negative scores for measures of multiple domains of body image [3,10-13]. Consequently, BMI appears to be the predominant measure of obesity used in studies examining the impact of overweight/obesity on body image [10,12,14].

Researchers have however suggested other means of quantifying overweight/obesity status in body image research, including skinfold assessment and bioelectrical impedance analysis [15]. However, these methods are more time consuming, have pre-test guidelines and may need trained scientists to complete in the case of skinfold analysis [15]. While the assessment of weight status via BMI is quick, non-labour intensive and does provide a measure of overall weight status for population monitoring [16], its use as a means to quantify overweight/obesity has been questioned in general [17] and specifically in the context of body image research [15]. This issue is exacerbated when examining this relationship in children and adolescents.

The validity of BMI as a measure of adiposity has been based on the assumption that as BMI increases so does adiposity [17]. However, studies have documented strong evidence of curvature in the the assumption of a linear body fat – BMI relationship [18,19]. Such curvature is not trivial and research has demonstrated that a one unit increase in BMI for a group of thin women from 15 to 16 (kg/m^2^) represents an increase of 2.3% body fat whereas, for obese women (35 to 36 kg/m^2^), a one unit increase represents only a 0.3% increase in body fat [20]. Consequently, if linearity is assumed and curvature ignored in this relationship, the true association between BF% and BMI will be systematically underestimated in people with lower values of BMI and overestimated in people with higher BMI values [20]. Moreover, when considering BMI in children BMI is unlikely to be normally distributed [21,22]. Thus, when BMI is used in statistical analysis, as is often the case, assumptions of normality are violated and inferences made cannot be trusted [22].

Studies have however proposed, an alternative measure, named variously as lean or inverse body mass index (iBMI, cm^2^/kg) as a more suitable proxy for body fatness in epidemiological research [21-23]. IBMI has a biologically sound basis and better reflects lean mass alongside non-lean mass (e.g., fat mass) compared to BMI [22]. The suggestion that iBMI is more biologically sound in comparison to BMI comes from the work of Nevill and Holder [22]. Although a comprehensive explanation of this issue is beyond the scope of the present paper See [22] for a full overview, in brief BMI as a measure of overweight and obesity arose through the combination of height and body mass via multiple regression to fit an implied model which is not based on any biological principles [22]. However, lean body mass is more stature related than weight. Therefore, a better model to predict adiposity should employ a measure of leanness (as is the case with iBMI) as it is based on biological principles rather than an empirically derived model as is the case with BMI [22].

This iBMI has been found to be normally distributed and a better predictor of adiposity in adults [20,22]. BMI is less likely to be normally distributed in children [22] and exhibits a curvilinear relationship with body fatness [24]. Given the range of studies that have used BMI as the sole measure of overweight/obesity when examining the body image- overweight/obesity relationship, an examination of the utility of iBMI in describing relations between body image and overweight/obesity status in children and young adolescents would seem meritous.

This is particularly so given the mixed nature of studies that have examined the relations between BMI and various measures of body image in the literature. Some studies identify that higher BMI is associated with more negative body image or increased body dissatisfaction [10-12] but that this pattern may differ across different ethnic [25] or gender [3,26] groups. Prior research has also identified that in the context of the BMI-mortality relationship there is an ‘optimum’ survival rate when BMI is in the mid range of values where all-cause mortality is minimised [27]. These authors also identified that modelling this association using BMI was also problematic in terms of fitting this to a quadratic and suggested that studies should seek to invert BMI in order to examine this issue in future research [27].

As body image concerns have been consistently associated with overweight and obesity and are seen as an important health related variable, this study sought to build on suggestions of prior authors [21-23,27] by comparing the utility of either iBMI or BMI to optimise the relationship with body esteem in young adolescents.

## Methods

### Study sample

Seven hundred and fifty six young adolescents (394 boys and 362 girls) participated in the study (mean age *± SD* was 11.4 *±* 1.64 years). Participants were from black (*n* = 62), white (*n* = 549) and South Asian (*n* = 145) ethnic groups. Participants were selected from secondary schools (n = 5) within the City of Birmingham using cluster sampling. Informed consent was provided by parents/guardians and the adolescents themselves. The study was approved by the institutional ethics committee of Coventry University.

### Measures

Body Esteem: The body esteem scale for children, a self-report measure designed to measure self evaluations of one’s body [28,29] and reflects the attitudinal component of body image [30] was used to assess body esteem. This measure comprises yes/no responses to 24 items such as ‘I wish I were thinner’. The minimum possible score is 0, reflecting low body esteem and the highest possible score is 24, reflecting high body esteem. This scale has previously been used to assess body esteem in children and adolescents [28,29] and acceptable psychometric properties have been established [28]. Furthermore, in a pilot group of thirty, 11 year olds (the age of the youngest participants in the study) values for two week test re-test reliability (*R* = .83) were acceptable as were scores for Cronbach’s alpha (*α* = .90).

Overweight/Obesity Status: Following completion of the body esteem scale, participants’ height (cm) and mass (kg) were measured using a Seca stadiometer and weighing scales (Seca Instruments Ltd, Germany). From this BMI was determined as kg/m^2^ and iBMI was determined as cm^2^/kg. In the current study these were both considered as measures of body shape in line with Mosimann’s definition of body shape as the ratio of two body dimensions measured in the same units that yield a ‘dimensionless’ ratio variable [31].

### Statistical analysis

Data were analysed for both BMI and iBMI using a 2 (gender) X 3 (ethnicity) ways ANCOVA controlling for iBMI and iBMI^2^ and repeated using BMI and BMI^2^ as covariates. As prior research had identified gender and ethnicity as relevant variables in the relationship between overweight and obesity and measures of body image, this method enabled any differences in body esteem between gender and ethnic groups to be determined whilst at the same time establishing the amount of variance explained by the two measures of overweight/obesity status (BMI vs. iBMI) as both a linear and quadratic function. Subsequent analysis was also conducted for separate gender groups recognising that body image development is a specifically gendered phenomenon [26]. Data were also assessed for normality for separate gender groups using the Kolmogorov-Smirnov test and, in line with prior studies in this area [21,23]. Elementary differential calculus was also employed to determine the iBMI point at which body esteem scores peaked for the whole sample and for boys and girls separately. Statistical significance was set at 0.05 a priori and the Statistical package for Social Sciences (SPSS, Version 18, SPSS Inc., Chicago, Ill, USA) was used for all analysis.

## Results

**Figure 1:**
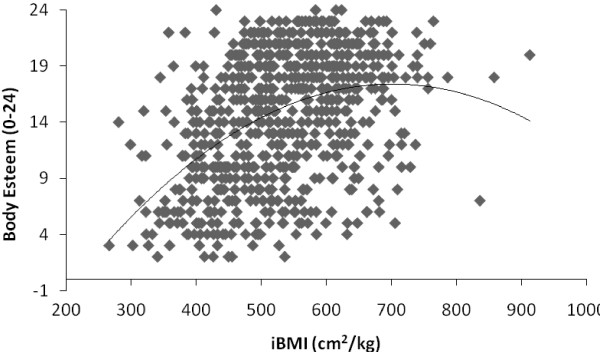
**The relationship between iBMI (cm**^**2**^**/kg) and Body Esteem in British adolescents.**

Results from ANCOVA for the pooled sample of boys and girls indicated that the relationship between body esteem and BMI was linear, i;e., the BMI^2^ was not significant when used as covariates (P > .05). In contrast, when iBMI and iBMI^2^ were used as covariates, both were significant (both P = .0001) a finding that suggests the association between iBMI and body esteem was curvilinear in nature (See Figure 1). Elementary differential calculus indicated that the peak or ‘optimal’ body esteem scores occured at iBMI = 700 cm^2^/kg. Moreover, iBMI was a better predictor of body esteem (Adjusted R^2^ = .213, P = .001) predicting 21.3% of the variance in body esteem compared to 20.5% in BMI (Adjusted R^2^ = .205, P = .001). Furthermore, results from the Kolmogorov-Smirnov test indicated that BMI was not normally distributed (*P* = .0001) but that iBMI was (*P* >0.05). Results from this analysis controlling for both BMI (P = .013) and iBMI (P = .001) also indicated significant gender X ethnicity interactions for body esteem whereby body esteem was significantly higher in boys compared to girls for white and black ethnic groups but was not significantly different between south Asian boys and girls.

**Figure 2:**
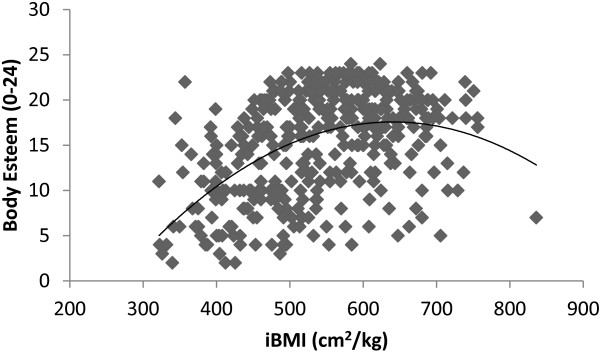
**The relationship between iBMI (cm**^**2**^**/kg) and Body Esteem in boys.**

**Figure 3:**
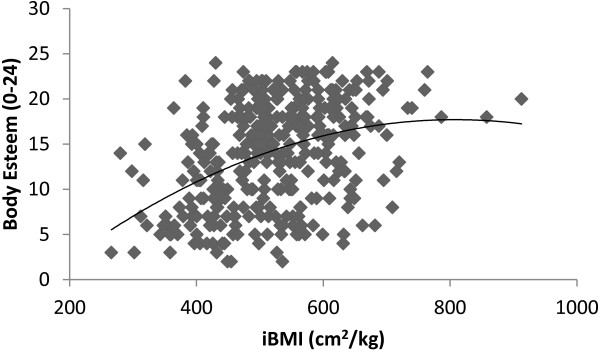
**The relationship between iBMI (cm**^**2**^**/kg) and Body Esteem in Girls.**

When considered as separate gender groups, the results were similar to those presented for the pooled sample. For both boys and girls BMI was not normally distributed (P = .0001 in both cases) whereas iBMI was (P = .09 for boys and .200 for girls). Furthermore, neither BMI nor BMI^2^ were significant when used as covariates for separate samples of boys and girls (P > .05) but when iBMI and iBMI^2^ were used as covariates both were significant (both P = .0001) for both gender groups. Similar to the data for the pooled sample, when gender specific slope parameter estimates were examined, data indicated that the association between iBMI and body esteem was quadratic and curvilinear in nature. However, the slope parameter estimates indicated that the nature of this curvilinear relationship was different for boys and girls. When taken as individual gender groups, the peak or ‘optimal’ body esteem scores were calculated as iBMI = 642 cm^2^/kg for boys (See Figure 2) and 800.64 cm^2^/kg for girls (See Figure 3). Likewise, similar to data for the whole sample, in boys, iBMI predicted significantly more of the variance in body esteem scores (Adjusted R^2^ = .250) predicting 25% of the variance in body esteem compared to 23% in BMI (Adjusted R^2^ = .230). However, in girls iBMI and BMI predicted a similar amount of the variance in body esteem scores (Adjusted R^2^ = .164 for both iBMi and BMI) predicting 16.4% of the variance in body esteem scores in both cases.

## Discussion

The present study adds to the data in this area by evidencing that iBMI is a better predictor of body esteem in young adolescents compared to BMI. BMI has been widely used as a measure of overweight/obesity status in prior research examining on body related concerns in children and young adolescents e.g., [9] often using parametric statistical techniques for analysis. This is despite BMI rarely being normally distributed in this population [22] and BMI evidencing a curvilinear relationship with body fatness in children and young adolescents [24]. The alternative measure of iBMI was subsequently proposed as an alternative to BMI to understand the association between health indices and overweight/obesity in young people [20,21]. The results presented here support prior studies which have identified iBMI as a better proxy measure of leanness in the leanness to overweight/obesity continuum than BMI [20,21] but extend the literature in this area by indentifying its applicability as a measure in body esteem research. Notably, this measure is no more difficult or labour intensive to calculate than BMI, it also has a sound biological basis as a measure of weight status and reflects the whole leanness to obesity continuum see [22] for a review. It is of course possible to statistically transform BMI data to ensure it is normally distributed and therefore can be used alongside parametric statistical techniques. However, such transformations do not address the issue of the biological basis of BMI as a measure of leanness to overweight/obesity whereas use of iBMI does.

What is of particular interest in the present study is that iBMI demonstrated a curvilinear relationship with body esteem in this group of young adolescents. iBMI has also been termed lean body mass index as it evidences a linear association with lean mass [22], the results of this study suggest that body esteem increases as lean mass also increases. This suggestion agrees with the assertions made previously by authors when explaining the relationship between BMI and various measures of body image in that higher BMI (and therefore assumed higher body fatness) is associated with poorer body image and conversely that lower BMI (and therefore assumed leanness) is associated with a more positive body esteem [4,7]. In the current study, the peak of the association between body esteem scores and iBMI was examined using elementary differential calculus, in line with methods suggested by Durazo-Arvizu et al. [27]. The body esteem - iBMI relationship was curvilinear so the assumption of a linear relationship as suggested by prior authors [4,7] only holds true only to an apex of approximately 700 cm^2^/kg. After this point even when lean mass increases body esteem scores begin to decline. Moreover, this curvilinear association held true for separate gender groups but the peak of this association differed for boys and girls with the peak of this relationship occurring at a value of 642.8 cm^2^/kg for boys and 800.64 cm^2^/kg for girls. These values could therefore be considered an ‘optimal’ in line with assertions previously made for other health related variables [27].

However, the different curves evidenced by boys and girls in the current study are important as the data indicate that the peak of body esteem scores occur at a lower degree of leanness for boys compared to girls. In lay terms this would indicate that the point at which body esteem scores are highest for girls is at a point of very high leanness to the point where, for girls body esteem peak at an equivalent BMI value of 12.5 kg/m^2^ (calculated as BMI = 10000/iBMI = 10000/800.64 = 12.5), a value which would classify them as overly ‘thin’ and at increased health risk according to international cut-off points [32]. Conversely, the peak of the body esteem – iBMI relationship for boys sits at a BMI equivalent of 15.5 kg/m^2^, a value within the ‘healthy’ BMI range for children and adolescents [16]. This is not however surprising given that an individual’s self-evaluation of their own body may be shaped by societal values of what an ‘attractive’ physique is [4,7] and the curvilinear relationship identified here likely reflects the western societal ideal of what an ‘ideal’ body is whereby individuals who are overly fat or overly lean do not conform to the ideal range of the ideal body and thus present lower body esteem. The different curves identified for boys and girls support prior assertions that body image development is a gendered phenomenon for reviews see [33] with the ideal physique for females being one which is super-thin [4] whereas the same does not hold true for males [34]. One potential reason why iBMI accounted for a greater proportion of body esteem than BMI in boys than girls may be because iBMI is a better reflection of leanness than BMI and, with boys, leanness may be more associated with body esteem at both high and low ranges of leanness compared to girls. This suggestion is however speculative.

No studies to date have reported this curvilinear relationship and thus future research is required which confirms the findings presented here. Despite this, the curvilinear nature of the iBMI – body esteem relationship would appear to support prior research that has identified high level of adiposity and or lean mass as being associated with poorer body esteem [7,10,31].

Whilst not the focus of the present study, this research also indicates that body esteem interacts with gender and ethnicity in young adolescents. In particular, body esteem scores were higher for boys from white and black ethnic groups, a finding which supports a range of prior research on this topic [8,9,11,34]. The data presented in this respect are not new and this finding should not be considered the major contribution of this brief report.

Despite this, the present study is not without its limitations. The study is cross-sectional in nature and therefore no comment can be made regarding cause and effect within the body esteem – overweight/obesity relationship (irrespective of whether BMI or iBMI is being considered). Furthermore, the body esteem scale as used in the present study provides a measure of global appearance satisfaction and only a minority of items within the questionnaire explicitly address weight, size and body shape. Future research may therefore be beneficial which examines the utility of iBMI in predicting more body weight/shape related measures of body image such as body areas satisfaction or overweight preoccupation. Also, given the age range of the participants in the present study, it is likely that participants included young adolescents across a range of maturational stages. Maturation was not assessed in the present study due to the sensitive nature of maturation assessment in young adolescents. This is important due to the changes in physique that accompany maturation. Such changes may mean that the relationship between body esteem and overweight/obesity varies throughout the process of growth and maturation and children move into and the out of adolescence. Future research would therefore benefit from including maturation analysis it in any subsequent study of this issue.

Although researchers could potentially analyse their BMI data in subgroups of participants, split into normal weight, overweight and obese groups to overcome the issue of normality with BMI, there may be times when researchers wish to examine this as a continuous variable to better understand the relationship between overweight and obesity and body esteem in young adolescents. In this case, the results of this study suggest that iBMI is a predictor of body esteem, comparable to BMI, and could be considered by researchers in future work.

## Conclusions

Prior studies have predominantly examined the association between overweight/obesity and body esteem in children and adolescents using BMI. The data presented here indicate that inverse body mass index or ‘iBMI’ may be an alternative useful measure of leanness which is better able to predict body esteem scores in young adolescents compared to BMI. This study also identifies that there is an optimum range of iBMI associated with more positive body esteem in this population. When translated to BMI this optimum equates to a value of 12.5 kg/m^2^ for girls and 15.5 kg/m^2^ for boys. From a public health perspective, practitioners and research may want to consider iBMI as an alternative measure of overweight and obesity in their research and should also consider the curvilinear relationship of the association between overweight and obesity and body esteem in this population when designing interventions aimed at reducing overweight and obesity and/or enhancing body image in young adolescents.

## Competing interests

The authors have no competing interests.

## Authors’ contributions

MD was responsible for study design, data collection, analysis and writing the manuscript, YAN was responsible for study design and data collection, AN was responsible for study design, analysis and writing the manuscript. All authors read and approved the final manuscript.

## Pre-publication history

The pre-publication history for this paper can be accessed here:

http://www.biomedcentral.com/1471-2458/13/662/prepub
